# Heart Rate Control with Landiolol Hydrochloride in Infants and Neonates During Cardiac Surgery

**DOI:** 10.1007/s00246-025-03824-6

**Published:** 2025-03-10

**Authors:** Matthias Müller, Lukas Andreas Puschmann, Thomas Zajonz, Martin Unger, Jakob Ackerl, Olga Shatilova

**Affiliations:** 1 Pediatric Heart Center Giessen, Department of Anesthesiology, Intensive Care Medicine, Pain Therapy University Hospital of Giessen and Marburg Gmbh, Giessen, Germany; 2 AOP Orphan Pharmaceuticals GmbH, Vienna, Austria

**Keywords:** Heart rate, Landiolol, Cardiac surgery, Pediatric

## Abstract

Perioperative tachyarrhythmias are common in children undergoing cardiac surgery, contributing to significant mortality and morbidity. Landiolol, an ultrashort-acting beta-blocker, offers selective heart rate (HR) control in pediatric patients with tachycardia during cardiac surgery. The LANDI-cardioPed study assessed landiolol's safety and efficacy in children during cardiac surgery. The retrospective review included patients under 1 year of age with intraoperative tachycardia during cardiac surgery treated with landiolol hydrochloride (Rapibloc®) at the Pediatric Heart Center Giessen, Germany (June 2017–October 2022). The primary endpoint was the proportion of patients achieving a target HR (< 160 bpm) by surgery end. Among 24 patients (46% neonates, 54% infants; mean weight: 4.8 kg; preexisting heart failure in 71%), 83% achieved the target HR by surgery’s end, with all patients reaching it during treatment. Median HR normalization time was 4.0 min and 75% of children reached it within 18.5 min. Landiolol demonstrated good hemodynamic stability. It reduced HR by 6% from baseline while maintaining stable blood pressure and HR/BP ratios, with no increased vasopressor use. The drug was titrated individually, with a mean initial dose of 23 µg/kg/min, the dose was 26 µg/kg/min upon reaching target HR, and the maximum dose of 49 µg/kg/min. The treatment duration averaged 10.5 h. Safety was favorable; three adverse events unrelated to landiolol were reported, and no dose interruptions occurred. Landiolol was effective and safe for HR control in neonates and infants with tachycardia during cardiac surgery, which aligns with current knowledge on its use in children.

**Supplementary Information:**

The online version contains supplementary material available at 10.1007/s00246-025-03824-6.

## Introduction

Narrow complex tachycardia (NCT) is a significant concern during and after pediatric cardiac surgery, affecting up to 10% of post-operative pediatric cardiac patients and associated with higher mortality rates and longer ICU stays [1] [2].

Risk factors include younger age, longer bypass and cross-clamp times and surgeries near the atrioventricular node [1] [3] [4] [5] [6]. Recent studies have shown that post-surgical atrial and ventricular septal defects patients exhibit abnormal heart rate (HR) variability patterns, indicating reduced parasympathetic activity and sympathetic predominance. These patterns are linked to increased morbidity and mortality across various patient populations and clinical conditions [7, 8].

In addition, tachycardia can be induced by inotropes and catecholamines frequently used during and after cardiac surgery with cardiopulmonary bypass (CPB) [9, 10]. Even with adequate intraoperative stress protection and avoidance of hypovolemia, anemia, and hyperthermia, sinus tachycardia and junctional ectopic tachycardia (JET) are frequently observed in neonates and infants undergoing congenital heart surgery, potentially leading to worse outcomes [11]. Hagel et al. have demonstrated that high HR and low blood pressure (BP) are associated with a poorer 7-day outcome following congenital heart surgery [12]. Consequently, HR control is a critical aspect of perioperative management in pediatric cardiac patients.

Treating tachycardia typically addresses two goals: terminating the arrhythmia/controlling HR and preventing its recurrence. Infusion of short-acting beta-blocker is an accepted pharmacological option to treat tachyarrhythmia, as it blocks sympathetic activity and effectively reduces HR [13] [14]. In the perioperative setting, highly selective beta-blockers are particularly valuable, as they minimally interfere with the positive inotropic and cardioprotective effects of the beta-2 receptors. For instance, intravenous esmolol (beta-1-selective blocker) has been shown to reduce HR and cardiac workload in neonates without compromising hemodynamics [15].

Landiolol, a novel highly cardio-selective beta-blocker (ß1/ß2 255:1), offers advantageous pharmacological properties for cardiac surgery [16]. Its short half-life (4 min), absence of tolerance, and limited negative inotropic effect make it particularly suitable [17] [18] [16]. Notably, landiolol demonstrates significantly higher beta-1 selectivity compared to esmolol [18].

Landiolol has been used to treat various forms of supraventricular tachycardia in pediatric population. Recent clinical studies in Japan confirmed its safety and efficacy in different age groups: one study involved 21 patients aged 1–13 months, most with congenital heart disease or perioperative tachyarrhythmia [19]; HEARTFUL study included 25 patients aged 3 months to 15 years with low cardiac function [20]. Based on the results, landiolol was approved in Japan to treat tachyarrhythmia (supraventricular tachycardia, atrial fibrillation and atrial flutter) in children with low cardiac function. The available clinical data suggests that children's response to landiolol in terms of HR and rhythm control is similar to that of adults [19–21]. Currently, landiolol is approved in the European Union (EU) for use in adults to treat supraventricular tachycardia and for the rapid control of ventricular rate in patients with atrial fibrillation or atrial flutter in perioperative or other circumstances, and for non-compensatory sinus-tachycardia.

The aim of the present study was to investigate the safety and effectiveness of landiolol in the treatment of tachycardia in neonates and infants undergoing cardiac surgery. The primary outcome was the percentage of patients achieving the target HR, defined as HR < 160 bpm, at the end of surgery. The HR threshold of 160 bpm was selected based on the established treatment criteria for antitachycardiac interventions in neonates and infants at our institution. This value corresponds to the 98th percentile of HR observed in healthy newborns [22, 23]. It is worth noting that neonates with complex congenital heart conditions, such as hypoplastic left heart syndrome or pulmonary outflow tract obstruction, typically exhibit HRs below 160 bpm postnatally [24]. This intervention threshold considers the specific conditions in the operating room, particularly right after patients are weaned off CPB following complex cardiac surgeries. In this setting, optimizing blood flow while minimizing myocardial oxygen consumption is critical, as it leads to favorable outcomes and underscores the concept of an ideal HR.

Secondary outcomes included: percentage of patients achieving target HR during landiolol treatment and observation period; time from landiolol treatment start until achievement of target HR (only for responders); percentage decrease in HR at the end of surgery; and change in inotropic drug usage during landiolol treatment; safety endpoints.

## Methods

This retrospective chart review examined all patients with tachycardia treated with landiolol hydrochloride at the Pediatric Heart Center Giessen from June 1, 2017, to October 12, 2022. We identified cases by querying our in-house patient data management system (PDMS, ICU-Data, IMESO® GmbH, Giessen, Germany) for pediatric cardiac surgery patients receiving landiolol treatment. Data management and statistical analysis were performed by independent contract research organizations.

### Inclusion Criteria

1. Patients under 1 year of age with tachycardia during cardiac surgery, who received landiolol.
2. Tachycardia onset after weaning from CPB.
3. HR ≥ 160 bpm at landiolol infusion initiation.

### Exclusion Criteria

1. Participation in investigational medicinal product studies.
2. Tachycardia due to uncontrolled bleeding.

### Observation Period and Treatment

We defined the observation period from landiolol treatment initiation during surgery until 60 min post-discontinuation, divided into four phases:

- Baseline: Surgery start to landiolol initiation.
- Intraoperative: Landiolol start to surgery end.
- Postoperative: Surgery end to landiolol discontinuation.
- Follow-up: 60 min post-landiolol discontinuation.

Landiolol, though not approved for pediatric use in the EU, was administered off-label via central venous catheter. We documented administration details (concentration, dose, infusion rate) and reasons for discontinuation.

### Data Collection and Analysis

Biometric and procedural data were collected. We defined tachycardia as HR ≥ 160 bpm and target HR as < 160 bpm. Hemodynamic parameters recorded included HR, invasive BP measurements, oxygen saturation, and body temperature. Vital signs were monitored at specific intervals throughout the observation period. We calculated the vasoactive-inotropic score (VIS) at multiple timepoints.

Adverse events (AEs) were classified, graded, and coded according to MedDRA Version 25.0.

We categorized patients into neonates (≤ 28 days) and infants (> 28 days) subgroups for descriptive analysis. AE causality assessments were conducted independently by the treating clinicians and reviewed during study data validation to ensure consistency and objectivity.

### Statistical Analysis

Statistical analysis included descriptive statistics for demographic data, baseline characteristics, and hemodynamic parameters. Categorical variables are presented as number and percentage, while continuous data are presented as mean ± standard deviation (SD). Wilson confidence intervals were calculated for the primary endpoint. Continuous vital sign parameters were assessed descriptively, p-values for significance of changes from baseline were determined using paired Student t-tests or Wilcoxon tests as appropriate. Fisher's exact test was used to detect differences between subgroups in an exploratory mode for testing. We considered a p-value < 0.05 significant and used SAS (version 9.4) for analysis and table generation.

Sensitivity analyses were conducted for the primary endpoint to confirm the robustness of the findings despite missing data. Missing values were tabulated separately and treated as non-achievement of the target HR in sensitivity analyses, ensuring transparency in reporting and interpretation.

### Ethical Considerations

The Justus Liebig University Giessen Ethics Committee approved this study (Project AZ 165/22), waiving informed consent due to its retrospective nature. We adhered to local data protection laws, anonymizing all patient information. The study design followed guidelines for good pharmacoepidemiology practices (GPP), STROBE guidelines, and applicable good clinical practice (GCP) principles. We ensured data integrity using a validated electronic case report form (eCRF) system.

## Results

### Patient Characteristics and Procedural Data

**Fig. 1:**
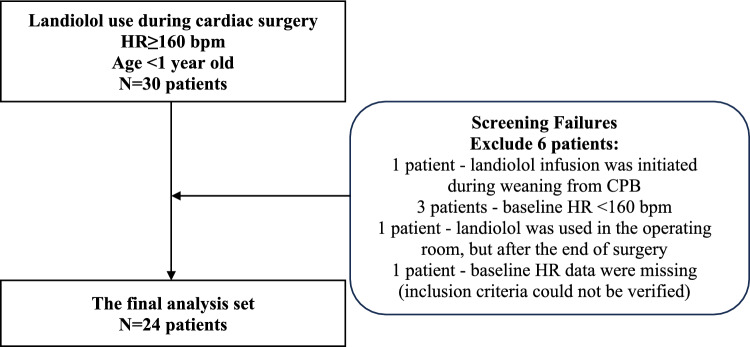
A flow chart of patient enrollment and exclusion from analysis

Thirty patients were initially screened, and six children were excluded from the analysis due to a screening failure (Fig. 1): for the first patient, the landiolol infusion was initiated during weaning from CPB rather than after separation from CPB as per protocol; three patients had baseline HR values below the inclusion threshold of 160 bpm; for one patient, intraoperative data on landiolol treatment were missing because the start of landiolol was recorded at the same time as the end of surgery; and one patient had no baseline HR values recorded, preventing confirmation of their eligibility based on inclusion criteria. The final cohort included 24 neonates and infants with intraoperative tachycardia during cardiac surgery with onset after weaning from CPB treated with landiolol.

Patient demographics are summarized in Table 1. The cohort had a mean age of 105.5 ± 97.6 days, with 54% infants and 46% neonates. Average body weight was 4.8 ± 1.4 kg. All patients underwent congenital heart surgery, with 33% having prior cardiac procedures. Preexisting heart failure was reported in 71% of patients (53% compensated, 47% decompensated). Patients underwent various types of heart surgeries, with 92% (22/24) accounting to high-risk surgeries (risk category ≥ 3) according to RACHS-1 (for heart transplantation, RACHS-2 was used). Mean surgical duration was 327 ± 100 min with CPB and aortic cross-clamp times of 182 ± 79 and 90 ± 50 min, respectively (SuppInfo Table 1).

**Table 1:** Demographic characteristics, overall and by age (FAS)

	Statistics	Neonate (≤ 28 days) N = 11	Infant (> 28 days) N = 13	Overall N = 24	Subgroup difference p-value
Age at treatment start [days]	n / missing	11 / 0	13 / 0	24 / 0	< .0001^3^
	Mean (± SD)	15.9 (± 8.53)	181.3 (± 67.96)	105.5 (± 97.61)	
Gender	n / missing	11 / 0	13 / 0	24 / 0	1.0000^1^
Female	n (%)	5 (45.5%)	5 (38.5%)	10 (41.7%)	
Male	n (%)	6 (54.5%)	8 (61.5%)	14 (58.3%)	
Weight [kg]	n / missing	11 / 0	13 / 0	24 / 0	< .0001^2^
	Mean (± SD)	3.60 (± 0.548)	5.83 (± 1.060)	4.81 (± 1.416)	
Prior existing heart failure	n / missing	11 / 0	13 / 0	24 / 0	0.6591^1^
No	n (%)	4 (36.4%)	3 (23.1%)	7 (29.2%)	
Yes	n (%)	7 (63.6%)	10 (76.9%)	17 (70.8%)	
Prior heart failure status	n / missing	7 / 0	10 / 0	17 / 0	1.0000^1^
Compensated	n (%)	4 (57.1%)	5 (50.0%)	9 (52.9%)	
Decompensated	n (%)	3 (42.9%)	5 (50.0%)	8 (47.1%)	
Heart rate [bpm]	n / missing	11 / 0	13 / 0	24 / 0	0.0258^2^
	Mean (± SD)	172.0 (± 6.02)	166.5 (± 5.08)	169.0 (± 6.09)	
Active pacemaker	n / missing	10 / 1	13 / 0	23 / 1	1.0000^1^
No	n (%)	9 (81.8%)	11 (84.6%)	20 (83.3%)	
Yes	n (%)	1 (9.1%)	2 (15.4%)	3 (12.5%)	
Prior relevant surgical history	n / missing	11 / 0	13 / 0	24 / 0	0.0020^1^
No	n (%)	11 (100%)	5 (38.5%)	16 (66.7%)	
Yes	n (%)	0	8 (61.5%)	8 (33.3%)	
Chronic beta blockade	n / missing	11 / 0	13 / 0	24 / 0	0.5761^1^
No	n (%)	2 (18.2%)	1 (7.7%)	3 (12.5%)	
Yes	n (%)	9 (81.8%)	12 (92.3%)	21 (87.5%)	
Mean arterial pressure [mmHg]	n / missing	11 / 0	13 / 0	24 / 0	0.6108^2^
	Mean (± SD)	58.4 (± 9.50)	56.4 (± 9.17)	57.3 (± 9.17)	

N = total number of patients per analysis group

n / missing = count of patients /number of missing observations

^1^Fisher's exact test; ^2^Two sample Welch's t-test; ^3^Two sample Wilcoxon rank-sum test

### Concomitant Medication

In all patients an opioid-based anesthesia, which was supplemented with intermitted boluses of midazolam and a continuous infusion of dexmedetomidine at a rate of 1 µg/kg/h. Sufentanil was administered at recommended doses of 0.2–1 µg/kg, with a median total dose of 20–30 µg per procedure depending on patient age, weight, and surgery duration. When remifentanil was chosen (3 patients), it was administered at a rate of 0.1–0.6 µg/kg/min.

After weaning off CPB all patients required inotropic and vasoactive support. Milrinone (0.5–1 µg/kg/min) and norepinephrine (up to a mean maximum dose of 0.53 µg/kg/min) were chosen as the primary agents for vasoactive and inotropic support due to their lesser effects on HR compared to dopamine, dobutamine and epinephrine (see also Vasoactive-Inotropic Score below).

Amiodarone was administered to three patients in the postoperative period and therefore had no effect on the primary outcome of the study. Esmolol was also administered postoperatively to nine patients, primarily for ongoing HR and rhythm management. Transitions were typically gradual, with esmolol initiated either immediately or shortly after landiolol discontinuation. The primary indication for switching was physician preference or protocol familiarity, not lack of efficacy.

### Hemodynamic Monitoring

**Fig. 2:**
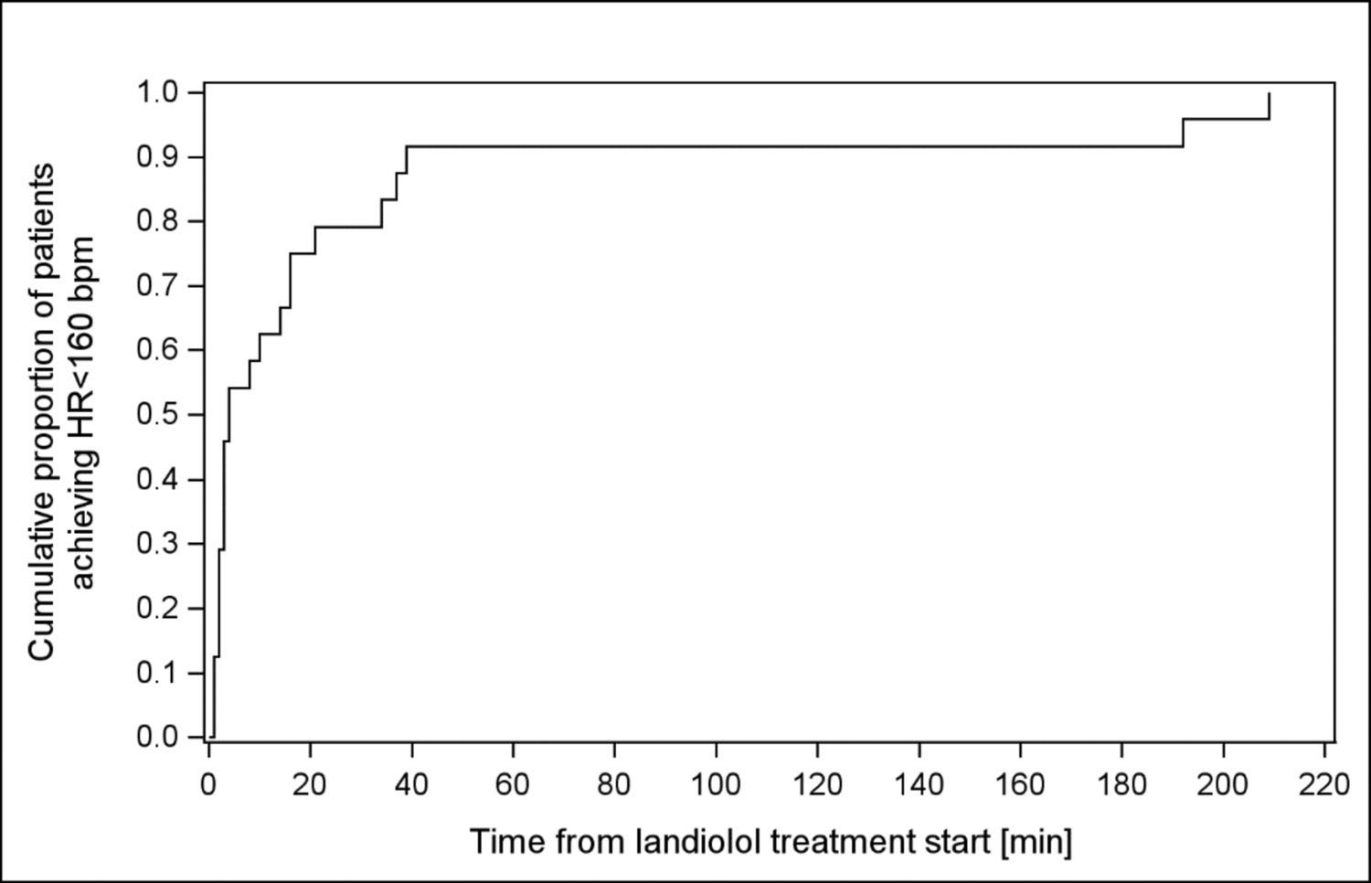
Kaplan—Meier curve for the time to achievement of target HR overall (FAS)

Prior to the start of landiolol infusion (baseline), patients exhibited a mean ± SD HR of 169.0 ± 6.1 bpm and a mean ± SD arterial blood pressure (MAP) of 57.3 ± 9.2 mmHg (Table 1). The target HR (< 160 bpm) was achieved in 83% (20/24) of patients by the end of surgery and in all patients during landiolol treatment (Fig. 2, Table 2). The mean time to target HR was 26.1 ± 55.0 min with the median time of 4.0 min (1.0–209.0).

**Table 2:** Results of study endpoints

Study endpoints	Study results
Primary endpoint	
- Percentage patients achieving target HR (< 160 bpm) until the end of surgery, N (%)	20/24 (83.3%)
Secondary endpoints	
- Percentage of patients achieving target HR (< 160 bpm) during landiolol treatment, N (%)	24/24 (100%)
- Percentage of patients achieving target HR (< 160 bpm) during observation period, N (%)	24/24 (100%)
- Time from landiolol treatment start until achievement of target HR (only in those patients who achieve heart target rate during the study), min	Mean (± SD): 26.1 (± 55.0) Median (min; max): 4.0 (1; 209)
- Percentage decrease in HR at the end of surgery, %	6.3%

Landiolol administration resulted in a statistically significant (p ≤ 0001) HR reduction at all evaluated time points except at 30 min after treatment initiation, at which data was missing for the major part of the study population (11/24 patients) (SuppInfo Table 2). At first target HR achievement, HR decreased by 17 ± 10 bpm (10%) from baseline to 153 ± 7 bpm (p < 0.0001). By the end of surgery, mean HR had decreased by 11 ± 18 bpm (6%) to 158 ± 18 bpm.

Blood pressure parameters (systolic, diastolic, and MAP remained stable throughout the observation period. No significant changes in MAP or systolic blood pressure (SBP) were observed during the landiolol treatment. When target HR was first achieved, mean MAP decreased from 57 ± 9 mmHg at baseline to 53 ± 14 mmHg but recovered to 61 ± 11 mmHg by the end of the surgery. SBP followed a similar trend (SuppInfo Table 3).

**Table 3:** List of reported adverse events

Age (days)	Underlying cardiac disease	Surgery performed	Adverse Event	Action(s) taken and autcome
154	- Complete atrioventricular septal defect - Transposition of the great arteries - Pulmonary atresia - TAPVR - Post AP-Shunt placement	TAPVR Repair	***Cardiovascular event:*** biventricular heart failure with low cardiac output 15 min after weaning off CPB	Veno-Arterial ECMO Recovered
27	- High degree aortic stenosis persisting after balloon valvuloplasty - Preoperative reduced left ventricular function - Endocardial fibroelastosis	Aortic valvuloplasty	***Cardiovascular event:*** pulmonary hypertensive crisis with right heart failure 40 min after weaning off CPB	Veno-Arterial ECMO Recovered
9	- CoA with hypoplastic aortic arch - Borderline left ventricle - ASD	CoA and Aortic Arch Repair, Partial Closure of the ASD	*Arrhythmia:* persisting JET and "dry tamponade" leading to cardiac arrest 775 min after weaning off CPB	CPR / Re-sternotomy / Veno-Arterial ECMO Recovered

*AP* Aortopulmonary, *ASD* Atrial septal defect, *CoA* Coarctation of the aorta, *CPB* cardiopulmonary bypass, *CPR* cardiopulmonary resuscitation, *ECMO* Extra Corporeal Membrane Oxygenation, *JET* junctional ectopic tachycardia, *TAPVR* Total anomalous pulmonary return

### Shock Index and Vasoactive-Inotropic Score

**Fig. 3:**
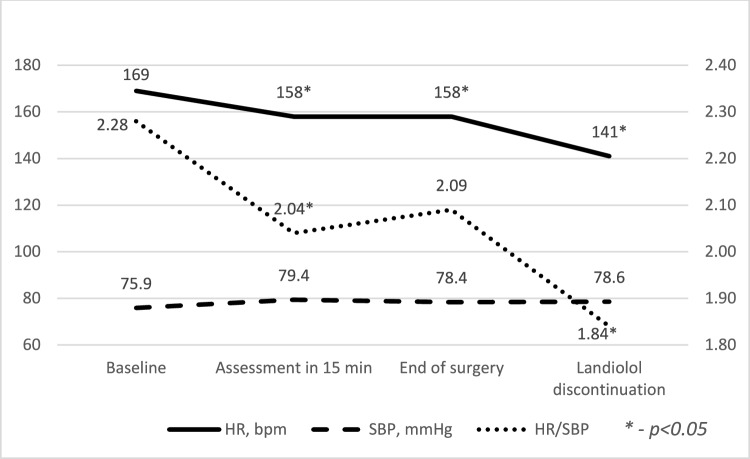
Changes over time in mean HR, SBP, and HR/SBP ratio during landiolol treatment

The reduction in HR coupled with stable BP led to a significant decrease in HR/SBP ratio, commonly referred to as the shock index, 15 min after landiolol infusion and at treatment discontinuation (Fig. 3). The mean ± SD HR/SBP ratio, decreased from 2.28 ± 0.3 at baseline to 2.04 ± 0.3 after 15 min of treatment, was 2.09 ± 0.4 at the end of surgery, and further decreased to 1.84 ± 0.3 upon landiolol withdrawal (SuppInfo Table 4).

The mean Vasoactive-Inotropic Score (VIS) showed minimal fluctuation: 19.0 ± 7.0 before landiolol administration (20/24 patients), 18.3 ± 6.2 during surgery (21/24 patients), 19.3 ± 7.6 during postoperative treatment (20/24 patients), and 19.3 ± 7.6 shortly after landiolol discontinuation (20/24 patients) (SuppInfo Table 6).

### Subgroup Analysis

Evaluation of the primary endpoint demonstrated similar findings in both age subgroups with no statistically significant differences in HR and BP responses (SuppInfo Table 2 and Table 3). However, subgroup analysis revealed a higher postoperative increase in mean VIS in neonates (19.7 ± 8.0) compared to infants (18.8 ± 7.4) (p = 0.044) (SuppInfo Table 6).

### Landiolol Administration

The mean duration of landiolol infusion was 10.5 ± 8.8 h, with a median of 8.7 h (range: 0.9—25.8 h). Postoperative administration was significantly longer than intraoperative administration with mean durations of 10.3 (± 8.8) hours and 0.6 (± 0.3) hours, respectively.

The initial mean landiolol dose was 23 ± 9 µg/kg/min with median 20 µg/kg/min (range: 9–54 µg/kg/min). Upon first achieving target HR, the mean dose increased slightly 26 ± 10 μg/kg/min. For continued HR control, the dose was up-titrated to 37 ± 13 μg/kg/min at the end of surgery and to 35 ± 12 μg/kg/min at discontinuation (SuppInfo Table 5).

The overall maximum landiolol dose was 49 (± 26) µg/kg/min. Intraoperatively, the maximum dose averaged 37 (± 14) µg/kg/min (median: 40 µg/kg/min; range: 18–62 µg/kg/min). Postoperatively, the mean maximum dose increased to 50 (± 26) µg/kg/min (median 40.8 µg/kg/min (range 35.7–163.0 µg/kg/min). The highest reported dose, 163.0 µg/kg/min, was well-tolerated by the infant during the 47-min infusion (7 min intraoperatively and 40 min postoperatively) before being reduced.

### Adverse Events

Three AEs were observed in three patients (3/24, 12.5%), with no fatalities. These AEs included one case each (1/24, 4.2%) of congestive cardiac failure/low cardiac output syndrome, acute right ventricular failure, and arrhythmia requiring cardiopulmonary resuscitation (CPR) and extracorporeal membrane oxygenation (ECMO). All AEs were classified as serious and life-threatening, but unrelated to the study drug. Complete recovery was achieved in all affected patients. Landiolol dosing remained unchanged in two patients, for one patient this documentation is missing. It was stopped in one patient after decreasing HR to 125 bpm while on ECMO support (Table 3, and SuppInfo Table 7).

## Discussion

The LANDI-cardioPed study represents the first evaluation of landiolol treatment in pediatric cardiac surgery patients. Our findings demonstrate that landiolol effectively and safely reduces HR in neonates and infants during cardiac surgery and early postoperative period.

Landiolol´s ultrashort action ensures rapid therapeutic effect, precise titration control, and quick recovery after drug discontinuation [19, 25–27]. Its high selectivity for beta-1-adrenergic receptors reduces catecholamine-induced tachycardia without significant negative inotropic effects [19]. In our study, the target HR (< 160 bpm) was achieved in most patients by the end of surgery and in all patients during landiolol treatment. The median time to target HR was 4.0 min, consistent with previous studies [27] [28]. Importantly, BP remained stable throughout the observation period without increased vasopressor requirements, confirming landiolol's minimal impact on BP and inotropy [29] [17].

Tachycardia-induced reduced organ perfusion in pediatric cardiac surgery contributes to increased postoperative morbidity and extended ICU stays [30]. Our clinical approach emphasizes pathophysiology directed therapy to prevent postoperative complications. The pharmacological strategy for CPB weaning following complex congenital cardiac procedures at our center employs a protocol of milrinone, combined with noradrenaline [31]. This regimen intentionally excludes traditional catecholamines (dopamine, dobutamine, and epinephrine) from the first-line therapy, aiming to achieve positive inotropic support while minimizing chronotropic effects. The mechanism of this combination is designed to maintain therapeutic efficacy even in the presence of selective beta-1-adrenoceptor antagonists, such as landiolol. Controlling HR after weaning off CPB to achieve “best heart rate” reduces oxygen consumption and improves oxygen delivery to the heart, which may be particularly important in cyanotic patients [31].

The HR/SBP ratio considered an additional parameter of ventricular function and independent predictors of adverse outcomes following congenital heart surgery [12]. In our high-risk patient cohort (92% RACHS-1 categories 3–6), landiolol consistently reduced HR/BP ratio, potentially decreasing the risk of adverse surgical outcomes. This aligns with findings that a HR/SBP > 1.83 (> 2.48 for neonates) is significantly associated with adverse events in the first 7 days post-pediatric cardiac surgery [12].

The study found no statistical difference between the subgroups for any parameter except postoperative VIS increase. The higher postoperative VIS in the neonatal subgroup compared to infants could be explained by higher percentage of patients undergoing high-risk procedures (in the neonatal subgroup, 100% patients were classified as RACHS-1 category ≥ 3 vs 85% in the infant’s subgroup). Neonates often need more intensive hemodynamic support due to immature myocardial function and limited ability to respond to volume or pressure changes, necessitating higher doses of inotropes and vasopressors. Furthermore, neonates are more sensitive to changes in vascular resistance, further contributing to the need for increased vasoactive support. [7] [32, 33].

The administered doses of landiolol were similar to those recommended for adults. Our higher starting dose (mean 23 ± 9 μg/kg/min) allowed rapid achievement of the target HR, with a mean dose of 26 ± 10 μg/kg/min observed at first HR < 160 bpm occurrence. Mean dose was 37 ± 13 μg/kg/min at the end of surgery, which is consistent with findings from the recent LANDI-PED study [34], which showed increased HR responses with higher landiolol doses, peaking at 40 μg/kg/min.

With regard to concomitant medications, recent studies have shown that adding central alpha-2 adrenergic receptor agonists such as dexmedetomidine to standard opioid-based anesthetic protocols does not significantly alter mean HR in infants undergoing complex congenital heart surgery [31]. This lack of chronotropic effect may be explained by the autonomic nervous system dysfunction commonly observed in congenital heart disease patients, particularly following prolonged CPB procedures [35, 36]. Given this evidence and the standardized anesthetic approach used across our cohort, we consider it unlikely that opioid administration had a significant impact on the HR outcomes observed in our study.

Landiolol was well-tolerated at a median maximum intraoperative dose of 40 μg/kg/min (range: 18–62 μg/kg/min). Only three adverse events (13%) were reported, all unrelated to the study drug and with full patient recovery. Overall, landiolol demonstrated good tolerability in neonates and infants with intraoperative tachycardia.

## Limitations

This study has several limitations. First, its retrospective nature may introduce bias in data collection and analysis. Second, the target HR of 160 bpm was set based on our clinical experience to ensure hemodynamic control after weaning off CPB in neonates and infants, rather than on established guidelines. This arbitrary threshold may limit the generalizability of our findings. Additionally, although HR and BP reductions were observed during landiolol infusion, it is important to acknowledge that other clinical factors, such as adjustments in inotropic or anesthetic medications, may have influenced hemodynamic stability. The retrospective design limits our ability to completely isolate the effects of landiolol from these confounding variables.

## Conclusion

This retrospective analysis supports current knowledge on landiolol use in children, suggesting it effectively reduces HR with minimal impact on myocardial contractility and blood pressure. These characteristics make landiolol particularly valuable in managing pediatric patients during cardiac surgery, where maintaining hemodynamic stability is crucial. Further prospective studies are warranted to confirm these findings and establish optimal dosing regimens for different pediatric age groups and cardiac conditions.

## Supplementary Information

Below is the link to the electronic supplementary material.

Supplementary file1 (DOCX 66 KB)

## Data Availability

No datasets were generated or analysed during the current study.
