# Supplementary material for: Heart Rate Control with Landiolol Hydrochloride in Infants and Neonates During Cardiac Surgery
Source: Pediatr Cardiol. 2025 Mar 10;47(2):709–17. doi: 10.1007/s00246-025-03824-6 (PMC12855301; doi:10.1007/s00246-025-03824-6)
Supplement: Supplementary file 1 — Supplementary file1 (DOCX 66 KB) [file 246_2025_3824_MOESM1_ESM.docx]

**SUPPLEMENTARY INFORMATION**

# Titel

**Heart Rate Control with Landiolol Hydrochloride in Infants and Neonates During Cardiac Surgery**

Journal name: **Pediatric Cardiology**

# Authors

Matthias Müller^1^, Lukas Andreas Puschmann^1^, Thomas Zajonz^1^, Martin Unger^2^, Jakob Ackerl^2^, Olga Shatilova^2^

# Affiliations

^1^ Pediatric Heart Center Giessen, Department of Anesthesiology, Intensive Care Medicine, Pain Therapy University Hospital of Giessen and Marburg GmbH, Giessen, Germany.

^2^ AOP Orphan Pharmaceuticals GmbH, Vienna, Austria.

# Corresponding Author

Matthias Müller

Pediatric Heart Center Giessen, Department of Anesthesiology, Intensive Care Medicine, Pain Therapy University Hospital of Giessen and Marburg GmbH, Giessen, Germany. Email: [matthias.f.mueller@chiru.med.uni](mailto:matthias.f.mueller@chiru.med.uni)-[giessen.de](http://giessen.de/). Tel.: +49-641-985-56550.

Table 1. Surgery details, overall and by age (FAS)

|  | Statistics | Neonate  (≤28 days) N = 11 | Infant (>28 days) N = 13 | Overall N = 24 |
| --- | --- | --- | --- | --- |
| Type of surgery* | n / missing | 11 / 0 | 13 / 0 | 24 / 0 |
| ASD creation/enlargement | n (%) | 0 | 2 (15.4%) | 2 (8.3%) |
| ASD repair, Patch | n (%) | 1 (9.1%) | 2 (15.4%) | 3 (12.5%) |
| ASD repair, Primary closure | n (%) | 4 (36.4%) | 0 | 4 (16.7%) |
| AVC repair, Complete | n (%) | 0 | 2 (15.4%) | 2 (8.3%) |
| Anomalous systemic venous connection repair | n (%) | 0 | 1 (7.7%) | 1 (4.2%) |
| Aortic arch repair | n (%) | 0 | 4 (30.8%) | 4 (16.7%) |
| Arterial switch operation | n (%) | 4 (36.4%) | 3 (23.1%) | 7 (29.2%) |
| Bidirectional cavopulmonary anastomosis | n (%) | 0 | 2 (15.4%) | 2 (8.3%) |
| Cardiac tumor resection | n (%) | 1 (9.1%) | 0 | 1 (4.2%) |
| Coarctation repair, End to end | n (%) | 1 (9.1%) | 1 (7.7%) | 2 (8.3%) |
| Coarctation repair, End to end, Extended | n (%) | 2 (18.2%) | 2 (15.4%) | 4 (16.7%) |
| Coarctation repair, Patch aortoplasty | n (%) | 0 | 3 (23.1%) | 3 (12.5%) |
| Congenitally corrected TGA repair, VSD closure and LV to PA conduit | n (%) | 0 | 1 (7.7%) | 1 (4.2%) |
| Damus-Kaye-Stansel procedure | n (%) | 0 | 1 (7.7%) | 1 (4.2%) |
| Norwood procedure | n (%) | 0 | 1 (7.7%) | 1 (4.2%) |
| PA debanding | n (%) | 0 | 6 (46.2%) | 6 (25.0%) |
| PA, reconstruction, Branch, Peripheral | n (%) | 0 | 1 (7.7%) | 1 (4.2%) |
| PA, reconstruction, Main | n (%) | 2 (18.2%) | 3 (23.1%) | 5 (20.8%) |
| PDA closure, Surgical | n (%) | 4 (36.4%) | 3 (23.1%) | 7 (29.2%) |
| PFO, Primary closure | n (%) | 1 (9.1%) | 2 (15.4%) | 3 (12.5%) |
| Pulmonary artery origin from ascending aorta repair | n (%) | 1 (9.1%) | 0 | 1 (4.2%) |
| Pulmonary venous stenosis repair | n (%) | 0 | 1 (7.7%) | 1 (4.2%) |
| RVOT procedure | n (%) | 1 (9.1%) | 1 (7.7%) | 2 (8.3%) |
| TOF repair, Ventriculotomy, Non-transanular patch | n (%) | 0 | 1 (7.7%) | 1 (4.2%) |
| TOF repair, Ventriculotomy, Transanular patch | n (%) | 0 | 1 (7.7%) | 1 (4.2%) |
| TOF repair, no ventriculotomy | n (%) | 0 | 1 (7.7%) | 1 (4.2%) |
| Transplant, Heart | n (%) | 0 | 1 (7.7%) | 1 (4.2%) |
| Truncus arterious repair | n (%) | 2 (18.2%) | 0 | 2 (8.3%) |
| VSD creation/enlargement | n (%) | 0 | 1 (7.7%) | 1 (4.2%) |
| VSD repair, Patch | n (%) | 4 (36.4%) | 3 (23.1%) | 7 (29.2%) |
| Valvuloplasty, Aortic | n (%) | 1 (9.1%) | 0 | 1 (4.2%) |
| Valvuloplasty, Mitral | n (%) | 1 (9.1%) | 1 (7.7%) | 2 (8.3%) |
| Valvuloplasty, Pulmonic | n (%) | 1 (9.1%) | 1 (7.7%) | 2 (8.3%) |
| Valvuloplasty, Tricuspid | n (%) | 1 (9.1%) | 1 (7.7%) | 2 (8.3%) |
| Length of surgery [min] | n / missing | 11 / 0 | 13 / 0 | 24 / 0 |
|  | Mean (±SD) | 283.3 (±68.26) | 363.5 (±109.23) | 326.7 (±99.59) |
|  | Median | 302.0 | 356.0 | 321.5 |
| Length of surgery [min] | Min; Max | 156; 376 | 198; 541 | 156; 541 |
| Cardioplegic solution | n / missing | 11 / 0 | 12 / 1 | 23 / 1 |
| Cristalloid cardioplegia | n (%) | 11 (100%) | 12 (92.3%) | 23 (95.8%) |
| Time to cardiopulmonary bypass from surgery start [min] | n / missing | 11 / 0 | 13 / 0 | 24 / 0 |
|  | Mean (±SD) | 77.7 (±22.13) | 97.5 (±31.83) | 88.5 (±29.04) |
|  | Median | 73.0 | 105.0 | 87.5 |
|  | Min; Max | 55; 128 | 53; 148 | 53; 148 |
| Duration of cardiopulmonary bypass [min] | n / missing | 11 / 0 | 13 / 0 | 24 / 0 |
|  | Mean (±SD) | 149.6 (±66.01) | 209.6 (±80.40) | 182.1 (±78.73) |
|  | Median | 156.0 | 208.0 | 184.0 |
|  | Min; Max | 48; 242 | 90; 341 | 48; 341 |
| Time to aortic cross-clamp from start of bypass [min] | n / missing | 11 / 0 | 13 / 0 | 24 / 0 |
|  | Mean (±SD) | 26.6 (±22.18) | 38.5 (±41.55) | 33.0 (±33.92) |
|  | Median | 17.0 | 22.0 | 18.0 |
|  | Min; Max | 9; 68 | 7; 150 | 7; 150 |
| Duration of aortic cross-clamp [min] | n / missing | 11 / 0 | 13 / 0 | 24 / 0 |
|  | Mean (±SD) | 81.3 (±52.10) | 98.2 (±48.29) | 90.4 (±49.70) |
|  | Median | 103.0 | 90.0 | 91.0 |
|  | Min; Max | 20; 154 | 43; 221 | 20; 221 |
| N = total number of patients per analysis group; n / missing = count of patients / number of missing observations *Percentage computed based on the number of patients in the analysis group. One patient could have more than one type of surgery. | | | | |

Table 2. Vital signs: Heart rate [bpm], absolute values and changes from baseline by visit and timepoint, overall and by age (FAS)

| **Visit** | **Value** | **Statistics** | **Neonate**  **(≤28 days) N = 11** | **Infant**  **(>28 days) N = 13** | **Overall N = 24** |
| --- | --- | --- | --- | --- | --- |
| Baseline | Absolute value | n / missing | 11 / 0 | 13 / 0 | 24 / 0 |
|  |  | Mean (±SD) | 172.0 (±6.02) | 166.5(±5.08) | 169.0 (±6.09) |
|  |  | Median | 173.0 | 167.0 | 168.5 |
|  |  | Min; Max | 161; 182 | 160; 175 | 160; 182 |
| Heart rate of <160bpm achieved for the first time | Absolute value | n / missing | 11 / 0 | 13 / 0 | 24 / 0 |
|  |  | Mean (±SD) | 152.5 (±6.25) | 152.6(±8.31) | 152.5 (±7.28) |
|  |  | Median | 153.0 | 157.0 | 156.0 |
|  |  | Min; Max | 143; 159 | 132; 159 | 132; 159 |
|  | Change from baseline | n / missing | 11 / 0 | 13 / 0 | 24 / 0 |
|  |  | Mean (±SD) | -19.5 (±8.12) | -13.8(±11.15) | -16.5 (±10.10) |
|  |  | Median | -20.0 | -9.0 | -15.5 |
|  |  | Min; Max | -29; -2 | -37; -3 | -37; -2 |
|  |  | p-value* | <.0001^1^ | 0.0002^2^ | <.0001^1^ |
| Regular assessment every 15 min (15 min) | Absolute value | n / missing | 8 / 3 | 10 / 3 | 18 / 6 |
|  |  | Mean (±SD) | 160.3 (±10.44) | 155.6(±7.23) | 157.7 (±8.85) |
|  |  | Median | 158.0 | 156.0 | 157.0 |
|  |  | Min; Max | 146; 182 | 143; 167 | 143; 182 |
|  | Change from baseline | n / missing | 8 / 3 | 10 / 3 | 18 / 6 |
|  |  | Mean (±SD) | -11.1 (±14.77) | -11.8(±9.94) | -11.5 (±11.93) |
|  |  | Median | -11.5 | -8.0 | -8.0 |
|  |  | Min; Max | -27; 15 | -32; -1 | -32; 15 |
|  |  | p-value* | 0.0706^1^ | 0.0045^1^ | 0.0008^1^ |
| End of surgery | Absolute value | n / missing | 11 / 0 | 13 / 0 | 24 / 0 |
|  |  | Mean (±SD) | 161.2 (±23.62) | 155.8(±11.47) | 158.3 (±17.86) |
|  |  | Median | 163.0 | 160.0 | 160.0 |
|  |  | Min; Max | 98; 188 | 125; 169 | 98; 188 |
|  | Change from baseline | n / missing | 11 / 0 | 13 / 0 | 24 / 0 |
|  |  | Mean (±SD) | -10.8 (±22.24) | -10.7(±13.26) | -10.8 (±17.52) |
|  |  | Median | -6.0 | -8.0 | -6.0 |
|  |  | Min; Max | -72; 12 | -50; 2 | -72; 12 |
|  |  | p-value* | 0.0703^2^ | 0.0015^2^ | 0.0001^2^ |
| Landiolol disconti-nuation | Absolute value | n / missing | 11 / 0 | 13 / 0 | 24 / 0 |
|  |  | Mean (±SD) | 146.8 (±13.50) | 135.7(±14.46) | 140.8 (±14.84) |
|  |  | Median | 147.0 | 132.0 | 141.5 |
|  |  | Min; Max | 123; 170 | 113; 159 | 113; 170 |
|  | Change from baseline | n / missing | 11 / 0 | 13 / 0 | 24 / 0 |
|  |  | Mean (±SD) | -25.2 (±17.59) | -30.8 (±13.76) | -28.2 (±15.54) |
|  |  | Median | -27.0 | -30.0 | -29.0 |
|  |  | Min; Max | -52; 9 | -50; -9 | -52; 9 |
|  |  | p-value* | 0.0008^1^ | <.0001^1^ | <.0001^1^ |
| Follow-up | Absolute value | n / missing | 11 / 0 | 13 / 0 | 24 / 0 |
|  |  | Mean (±SD) | 142.7 (±20.91) | 136.7 (±17.26) | 139.5 (±18.84) |
|  |  | Median | 149.0 | 137.0 | 143.5 |
|  |  | Min; Max | 86; 158 | 99; 162 | 86; 162 |
|  | Change from baseline | n / missing | 11 / 0 | 13 / 0 | 24 / 0 |
|  |  | Mean (±SD) | -29.3 (±22.32) | -29.8 (±17.34) | -29.5 (±19.33) |
|  |  | Median | -26.0 | -31.0 | -27.0 |
|  |  | Min; Max | -86; -3 | -61; 2 | -86; 2 |
|  |  | p-value* | 0.0010^2^ | <.0001^1^ | <.0001^1^ |
| N = total number of patients per analysis group   n / missing = count of patients / number of missing observations P-values are related to testing of statistical significance of percentage changes from baseline through  ^1^One sample Student's t-test or ^2^One sample Wilcoxon signed-rank test if normality assumption is violated | | | | | |

**Table 3. Vital signs: Systolic blood pressure [mmHg], absolute values and changes from baseline by visit and timepoint, overall and by age (FAS)**

| Visit | Value | Statistics | Neonate  (≤28 days) N = 11 | Infant (>28 days) N = 13 | Overall N = 24 | Subgroup difference p-value |
| --- | --- | --- | --- | --- | --- | --- |
| Baseline | Absolute value | n / missing | 10 / 1 | 12 / 1 | 22 / 2 | 0.44563 |
|  |  | Mean (±SD) | 78.2 (±14.31) | 74.0 (±10.00) | 75.9 (±12.03) |  |
|  |  | Median | 76.0 | 73.5 | 75.0 |  |
|  |  | Min; Max | 57; 104 | 59; 93 | 57; 104 |  |
| Heart rate of <160bpm achieved for the first time | Absolute value | n / missing | 10 / 1 | 12 / 1 | 22 / 2 | 0.58163 |
|  |  | Mean (±SD) | 72.2 (±13.03) | 75.3 (±13.09) | 73.9 (±12.85) |  |
|  |  | Median | 74.0 | 79.0 | 77.0 |  |
|  |  | Min; Max | 49; 89 | 59; 100 | 49; 100 |  |
|  | Change from baseline | n / missing | 9 / 2 | 11 / 2 | 20 / 4 | 0.61733 |
|  |  | Mean (±SD) | -4.6 (±8.25) | -2.3 (±11.77) | -3.3 (±10.14) |  |
|  |  | Median | -6.0 | -3.0 | -4.5 |  |
|  |  | Min; Max | -15; 8 | -22; 18 | -22; 18 |  |
|  |  | p-value* | 0.13611 | 0.53611 | 0.16191 |  |
| End of surgery | Absolute value | n / missing | 11 / 0 | 12 / 1 | 23 / 1 | 0.27583 |
|  |  | Mean (±SD) | 81.9 (±13.53) | 75.3 (±15.01) | 78.4 (±14.40) |  |
|  |  | Median | 77.0 | 78.0 | 77.0 |  |
|  |  | Min; Max | 68; 104 | 49; 102 | 49; 104 |  |
|  | Change from baseline | n / missing | 10 / 1 | 11 / 2 | 21 / 3 | 0.35904 |
|  |  | Mean (±SD) | 2.7 (±10.50) | 0.1 (±15.58) | 1.3 (±13.15) |  |
|  |  | Median | 1.5 | -3.0 | 0.0 |  |
|  |  | Min; Max | -14; 18 | -17; 41 | -17; 41 |  |
|  |  | p-value* | 0.43711 | 0.47462 | 0.64711 |  |
| Landiolol dis-continuation | Absolute value | n / missing | 11 / 0 | 13 / 0 | 24 / 0 | 0.88793 |
|  |  | Mean (±SD) | 79.1 (±13.46) | 78.2 (±16.08) | 78.6 (±14.62) |  |
|  |  | Median | 75.0 | 77.0 | 76.0 |  |
|  |  | Min; Max | 57; 100 | 49; 101 | 49; 101 |  |
|  | Change from baseline | n / missing | 10 / 1 | 12 / 1 | 22 / 2 | 0.84603 |
|  |  | Mean (±SD) | 3.1 (±18.38) | 4.5 (±14.11) | 3.9 (±15.79) |  |
|  |  | Median | 0.0 | 6.5 | 3.0 |  |
|  |  | Min; Max | -21; 35 | -21; 23 | -21; 35 |  |
|  |  | p-value* | 0.60661 | 0.29271 | 0.26411 |  |
| Follow-up | Absolute value | n / missing | 11 / 0 | 13 / 0 | 24 / 0 | 0.67643 |
|  |  | Mean (±SD) | 78.0 (±14.15) | 80.6 (±16.14) | 79.4 (±14.99) |  |
|  |  | Median | 74.0 | 76.0 | 75.5 |  |
|  |  | Min; Max | 57; 106 | 62; 111 | 57; 111 |  |
|  | Change from baseline | n / missing | 10 / 1 | 12 / 1 | 22 / 2 | 0.28043 |
|  |  | Mean (±SD) | -0.1 (±18.52) | 8.0 (±14.91) | 4.3 (±16.75) |  |
|  |  | Median | -2.0 | 7.0 | 1.0 |  |
|  |  | Min; Max | -25; 32 | -19; 38 | -25; 38 |  |
|  |  | p-value* | 0.98671 | 0.09011 | 0.24001 |  |
| N = total number of patients per analysis group; n / missing = count of patients / number of missing observations *P-values are related to testing of statistical significance of changes from baseline through  ^1^One sample Student's t-test or  ^2^One sample Wilcoxon signed-rank test if normality assumption is violated. ^3^Two sample Welch's t-test; ^4^Two sample Wilcoxon rank-sum test. | | | | | | |

Table 4. Vital signs: Heart rate and MAP ratio [bpm/mmHg], Heart rate and DBP ratio [bpm/mmHg], Heart rate and SBP ratio [bpm/mmHg], absolute values and changes from baseline by visit and selected timepoint, overall (FAS)

| **Visit** | **Value** | **Statistics** | **HR/MAP N = 24** | **HR/SBP N = 24** | **HR/DBP N = 24** |
| --- | --- | --- | --- | --- | --- |
| Baseline | Absolute value | n / missing | 24 / 0 | 22 / 2 | 22 / 2 |
|  |  | Mean (±SD) | 3.01 (±0.453) | 2.28 (±0.343) | 3.73 (±0.605) |
|  |  | Median | 2.96 | 2.22 | 3.63 |
|  |  | Min; Max | 2.1; 4.1 | 1.7; 3.1 | 2.5; 5.3 |
| Heart rate of <160bpm achieved for the first time | Absolute value | n / missing | 23 / 1 | 22 / 2 | 22 / 2 |
|  |  | Mean (±SD) | 3.05 (±1.114) | 2.15 (±0.445) | 3.45 (±0.719) |
|  |  | Median | 2.83 | 2.00 | 3.42 |
|  |  | Min; Max | 1.9; 7.5 | 1.4; 3.2 | 2.3; 5.1 |
|  | Change from baseline | n / missing | 23 / 1 | 20 / 4 | 20 / 4 |
|  |  | Mean (±SD) | 0.04 (±0.907) | -0.09 (±0.324) | -0.18 (±0.561) |
|  |  | Median | -0.06 | -0.08 | -0.18 |
|  |  | Min; Max | -1.2; 3.6 | -0.7; 0.6 | -1.6; 0.7 |
|  |  | p-value* | 0.4413^2^ | 0.2193^1^ | 0.1670^1^ |
| Regular assessment every 15 min (15 min) | Absolute value | n / missing | 18 / 6 | 17 / 7 | 17 / 7 |
|  |  | Mean (±SD) | 2.99 (±1.204) | 2.04 (±0.332) | 3.34 (±0.660) |
|  |  | Median | 2.65 | 1.96 | 3.16 |
|  |  | Min; Max | 2.0; 7.5 | 1.6; 2.9 | 2.7; 4.8 |
|  | Change from baseline | n / missing | 18 / 6 | 15 / 9 | 15 / 9 |
|  |  | Mean (±SD) | -0.05 (±0.975) | -0.18 (±0.267) | -0.29 (±0.293) |
|  |  | Median | -0.30 | -0.22 | -0.36 |
|  |  | Min; Max | -1.1; 3.6 | -0.5; 0.3 | -0.8; 0.3 |
|  |  | p-value* | 0.0432^2^ | 0.0200^1^ | 0.0020^1^ |
| End of surgery | Absolute value | n / missing | 24 / 0 | 23 / 1 | 23 / 1 |
|  |  | Mean (±SD) | 2.69 (±0.532) | 2.09 (±0.436) | 3.18 (±0.657) |
|  |  | Median | 2.73 | 2.09 | 3.02 |
|  |  | Min; Max | 1.5; 3.7 | 1.1; 3.0 | 1.9; 4.3 |
|  | Change from baseline | n / missing | 24 / 0 | 21 / 3 | 21 / 3 |
|  |  | Mean (±SD) | -0.32 (±0.593) | -0.13 (±0.393) | -0.49 (±0.733) |
|  |  | Median | -0.19 | -0.04 | -0.37 |
|  |  | Min; Max | -1.7; 0.7 | -1.2; 0.7 | -2.1; 0.6 |
|  |  | p-value* | 0.0133^1^ | 0.1410^1^ | 0.0062^1^ |
| Landiolol discontinuation | Absolute value | n / missing | 23 / 1 | 24 / 0 | 24 / 0 |
|  |  | Mean (±SD) | 2.35 (±0.359) | 1.84 (±0.302) | 2.82 (±0.443) |
|  |  | Median | 2.38 | 1.82 | 2.82 |
|  |  | Min; Max | 1.5; 2.9 | 1.2; 2.6 | 1.9; 3.7 |
|  | Change from baseline | n / missing | 23 / 1 | 22 / 2 | 22 / 2 |
|  |  | Mean (±SD) | -0.67 (±0.560) | -0.45 (±0.372) | -0.93 (±0.711) |
|  |  | Median | -0.89 | -0.43 | -1.04 |
|  |  | Min; Max | -1.8; 0.4 | -1.2; 0.2 | -2.7; 0.0 |
|  |  | p-value* | <.0001^1^ | <.0001^1^ | <.0001^1^ |
| N = total number of patients per analysis group; n / missing = count of patients / number of missing observations *P-values are related to testing of statistical significance of changes from baseline through  ^1^One sample Student's t-test or  ^2^One sample Wilcoxon signed-rank test if normality assumption is violated. ^3^Two sample Welch's t-test; ^4^Two sample Wilcoxon rank-sum test. | | | | | |

Table 5. Landiolol dose (µg/kg/min) by timepoint (FAS)

| **Timepoint** | **Statistics** | **Neonate**  **(≤28 days) N = 11** | **Infant (>28 days) N = 13** | **Overall N = 24** |
| --- | --- | --- | --- | --- |
| Dose initiated | n/ missing | 11 / 0 | 13 / 0 | 24 / 0 |
|  | Mean (±SD) | 21.64 (±7.395) | 24.08 (±10.825) | 22.96 (±9.299) |
|  | 95% CI for the mean | 16.67; 26.61 | 17.54; 30.63 | 19.04; 26.89 |
|  | Median | 20.00 | 19.67 | 20.00 |
|  | Min; Max | 9.7; 40.5 | 18.2; 54.8 | 9.7; 54.8 |
|  | | | | |
| Dose when heart rate of <160 bpm achieved for the first time | n/ missing | 11 / 0 | 13 / 0 | 24 / 0 |
|  | Mean (±SD) | 23.59 (±9.841) | 27.39 (±10.487) | 25.65 (±10.160) |
|  | 95% CI for the mean | 16.98; 30.20 | 21.05; 33.73 | 21.36; 29.94 |
|  | Median | 20.00 | 20.33 | 20.17 |
|  | Min; Max | 9.7; 43.2 | 18.2; 42.7 | 9.7; 43.2 |
|  | | | | |
| Dose at the end of surgery | n/ missing | 11 / 0 | 12 / 1 | 23 / 1 |
|  | Mean (±SD) | 36.74 (±15.238) | 37.33 (±11.037) | 37.05 (±12.905) |
|  | 95% CI for the mean | 26.51; 46.98 | 30.32; 44.35 | 31.47; 42.63 |
|  | Median | 40.00 | 39.42 | 39.50 |
|  | Min; Max | 19.3; 62.2 | 18.7; 60.0 | 18.7; 62.2 |
|  |  |  |  |  |
| Dose termination | n/ missing | 11 / 0 | 13 / 0 | 24 / 0 |
|  | Mean (±SD) | 37.61 (±5.677) | 32.51 (±15.466) | 34.84 (±12.064) |
|  | 95% CI for the mean | 33.79; 41.42 | 23.16; 41.85 | 29.75; 39.94 |
|  | Median | 40.00 | 37.50 | 39.00 |
|  | Min; Max | 26.5; 46.0 | 1.6; 60.0 | 1.6; 60.0 |
| N = total number of patients per analysis group  n / missing = count of patients / number of missing observations | | | | |

Table 6. VIS score: absolute values and changes from baseline, total and by time period, overall and by age group (FAS)

| **Time period** |  | **Statistics** | **Neonate (≤28 days) N = 11** | **Infant (>28 days) N = 13** | **Overall N = 24** | **Subgroup difference p-value** |
| --- | --- | --- | --- | --- | --- | --- |
| Over the whole study duration | Absolute value | n/ missing | 11 / 0 | 10 / 3 | 21 / 3 |  |
|  |  | Mean (±SD) | 20.15 (±7.918) | 18.92 (±7.660) | 19.56 (±7.625) | 0.7218^3^ |
|  |  | 95% CI for the mean | 14.83; 25.47 | 13.44; 24.40 | 16.09; 23.03 |  |
|  |  | Median | 21.06 | 16.93 | 20.32 |  |
|  |  | Min; Max | 9.0; 37.7 | 8.7; 32.9 | 8.7; 37.7 |  |
|  | | | | | | |
| Baseline time period | Absolute value | n/ missing | 10 / 1 | 10 / 3 | 20 / 4 |  |
|  |  | Mean (±SD) | 16.91 (±3.987) | 21.05 (±8.788) | 18.98 (±6.973) | 0.1988^3^ |
|  |  | 95% CI for the mean | 14.06; 19.76 | 14.76; 27.34 | 15.72; 22.24 |  |
|  |  | Median | 16.29 | 18.80 | 17.65 |  |
|  |  | Min; Max | 11.2; 22.3 | 9.7; 33.7 | 9.7; 33.7 |  |
|  | | | | | | |
| Intrao-perative time period | Absolute value | n/ missing | 11 / 0 | 10 / 3 | 21 / 3 |  |
|  |  | Mean (±SD) | 17.97 (±7.068) | 18.55 (±5.311) | 18.25 (±6.145) | 0.8332^3^ |
|  |  | 95% CI for the mean | 13.23; 22.72 | 14.75; 22.35 | 15.45; 21.05 |  |
|  |  | Median | 15.26 | 17.68 | 16.48 |  |
|  |  | Min; Max | 8.9; 30.3 | 10.2; 26.0 | 8.9; 30.3 |  |
|  | Change from baseline | n/ missing | 10 / 1 | 10 / 3 | 20 / 4 |  |
|  |  | Mean (±SD) | 1.911 (±6.2539) | -2.495 (±5.5325) | -0.292 (±6.1753) | 0.1127^3^ |
|  |  | 95% CI for the mean | -2.563; 6.385 | -6.453; 1.463 | -3.182; 2.598 |  |
|  |  | Median | 1.970 | -0.334 | 0.000 |  |
|  |  | Min; Max | -12.55; 10.18 | -13.86; 4.18 | -13.86; 10.18 |  |
|  |  | p-value* | 0.3592^1^ | 0.1876^1^ | 0.8347^1^ |  |
|  | | | | | | |
| Post-operative time period | Absolute value | n/ missing | 11 / 0 | 9 / 4 | 20 / 4 |  |
|  |  | Mean (±SD) | 19.65 (±8.040) | 18.82 (±7.408) | 19.28 (±7.570) | 0.8139^3^ |
|  |  | 95% CI for the mean | 14.25; 25.05 | 13.13; 24.52 | 15.74; 22.82 |  |
|  |  | Median | 21.13 | 17.13 | 18.98 |  |
|  |  | Min; Max | 8.2; 36.1 | 6.9; 29.4 | 6.9; 36.1 |  |
|  | Change from baseline | n/ missing | 10 / 1 | 9 / 4 | 19 / 5 |  |
|  |  | Mean (±SD) | 3.891 (±7.4892) | -2.540 (±5.2470) | 0.845 (±7.1530) | 0.0440^3^ |
|  |  | 95% CI for the mean | -1.466; 9.249 | -6.574; 1.493 | -2.603; 4.292 |  |
|  |  | Median | 3.659 | -1.788 | 1.054 |  |
|  |  | Min; Max | -11.26; 17.19 | -13.21; 5.75 | -13.21; 17.19 |  |
|  |  | p-value* | 0.1348^1^ | 0.1844^1^ | 0.6130^1^ |  |
| *A p-value to evaluate the significance of change from baseline:  ^1^ One sample Student's t-test; ^2^ One sample Wilcoxon signed-rank test. ^3^ Two sample Welch's t-test; ^4^ Two sample Wilcoxon rank-sum test. N = total number of patients per analysis group; n / missing = count of patients / number of missing observations. | | | | | | |

**Table 7. List of SAEs**

| **Patient ID** | **Age (days)** | **Weight (kg)** | **Heart Failure Status** | **Cardiac Diagnosis** | **Surgery Type** | **Landiolol Starting Dose (µg/kg/min)** | **Adverse Event** | **ECMO Required** | **Outcome** |
| --- | --- | --- | --- | --- | --- | --- | --- | --- | --- |
| **01-008** | 154 | 5.5 | Compensated | Infracardiac total anomalous pulmonary venous return, transposition of the great arteries, atrioventricular septal defect, pulmonary atresia | Reconstruction of the pulmonary arteries, creation of a central aortic pulmonary shunt, and total anomalous pulmonary venous repair | Starting from 18.17, stopped when heart rate reached 125/min while on ECMO | Life-threatening congestive heart failure/low cardiac output syndrome, echocardiography shows global hypokinesia 15 minutes after weaning off CPB, requiring second bypass run and ECMO therapy | Yes | Fully recovered |
| **01-018** | 27 | 3.1 | Decompensated | Critical valvular aortic stenosis, post aortic valve balloon dilation, endocardial fibroelastosis | Aortic valvuloplasty | Starting from 9.67 (increased to 38.67) | Life-threatening acute RV failure, 47 minutes after weaning off CPB requiring ECMO therapy, massive post-operative bleeding | Yes | Fully recovered |
| **01-022** | 9 | 3.0 | Compensated | Coarctation of the aorta, hypoplastic aortic arch, atrial septal defect | Extended end to end coarctation repair, aortic arch repair, atrial septal defect closure | Starting from 26.67 (increased to 60.0 then decreased to 40.0) | 11 hours after surgery, life-threatening hypotension requiring CPR, high dose epinephrine, arrhythmia, ECMO therapy | Yes | Fully recovered |
| CPB - cardiopulmonary bypass  ECMO - extracorporeal membrane oxygenation  CPR - cardiopulmonary resuscitation | | | | | | | | | |
